# An evaluation of preterm kidney size and function over the first two years of life

**DOI:** 10.1007/s00467-020-04554-y

**Published:** 2020-04-15

**Authors:** Yogavijayan Kandasamy, Donna Rudd, Eugenie R Lumbers, Roger Smith

**Affiliations:** 1 Department of Neonatology, The Townsville Hospital, 100 Angus Smith Drive, Douglas, Queensland 4814 Australia; 2 Mothers and Babies Research Centre, Hunter Medical Research Institute, HMRI, The University of Newcastle, Newcastle, NSW 2310 Australia; 3 College of Medicine and Dentistry, James Cook University, Douglas, QLD 4814 Australia; 4 College of Public Health, Medical and Veterinary Sciences, James Cook University, Douglas, QLD 4814 Australia; 5 School of Biomedical Sciences and Pharmacy, University of Newcastle, Newcastle, NSW 2310 Australia

**Keywords:** Preterm infant, Kidney volume, Estimated glomerular filtration rate

## Abstract

**Background:**

We carried out a study to determine the impact of prematurity on kidney development in the first 2 years of life.

**Methods:**

In this prospective study, extremely preterm neonates (gestation < 28 weeks) were recruited and underwent assessments at 6, 12, and 24 months of age. A cohort of neonates born term were also recruited and followed up for 24 months. The primary outcomes measured in this study were total kidney volume (TKV) and estimated glomerular filtration rate (eGFR); albuminuria and blood pressure measurements (all provided as mean (standard deviation)) were the secondary outcomes.

**Results:**

Fifty-three premature and 31 term neonates (control) were recruited. At the age of 24 months (corrected age), infants born preterm had significantly smaller TKV (56.1 (9.4) vs. 64.8 (10.2) mL; *P* = 0.006). There was no difference in eGFR. These preterm infants were smaller (11.25 (1.53) vs. 12.9 (1.8) kg; *P* = 0.002) and shorter (83.8 (3.0) vs. 86.3 (3.4) cm; *P* = 0.02) when compared with the control group. At 6, 12, and 18 months respectively, preterm infants had, relative to their height, significantly smaller kidney volumes (0.54 (0.1) vs. 0.59 (0.1) mL/cm, *P* = 0.05; 0.61 (0.1) vs.0.71 (0.1) mL/cm, *P* = 0.003; and 0.67 (0.1) vs.0.76 (0.1) mL/cm, *P* = 0.006).

**Conclusions:**

Relative to body length, TKV in premature infants is smaller. Since length reflects adult body proportions more accurately than BSA, TKV to height ratio may be a more important measure in the child. Despite smaller TKV (and therefore fewer nephrons), infants born prematurely achieve similar eGFRs in the first 24 months of life, probably due to single-nephron hyperfiltration.

## Introduction

Preterm birth accounts for 11% of births worldwide [1], and with increased investment in perinatal health among developing countries, the number of these infants surviving to adulthood is likely to increase [2]. Interventions such as antenatal steroids have improved survival [3], but the contribution of the oligonephropathy of prematurity [4] and its impact on the global burden of chronic kidney diseases (CKD) remain to be determined. The causes of CKD in ex-preterm infants are unknown, but there is evidence that prematurity leads to oligonephronia [4, 5].

In humans, nephrogenesis occurs in utero and is completed by 36 weeks of gestation. When an infant is born premature, normal nephrogenesis is interrupted [5]. Both animal [6] and human studies [7] indicate that ex-utero nephrogenesis continues for a short while after birth but a significant proportion of the new nephrons are abnormally developed. There is also data to indicate that prematurity changes the activity of the renin-angiotensin system [8].

The use of potentially nephrotoxic (yet clinically necessary) medications such as aminoglycosides, as well as sepsis, compounded by difficulties in detecting glomerular injury and kidney impairment, results in further nephron loss [9]. Brenner et al. [10, 11] demonstrated in animal models that single-nephron GFR increases to compensate for a reduction in the number of nephrons or when there are increased metabolic requirements. This compensatory increase is a response to the need to eliminate toxins and the end products of metabolism. Increased filtration per nephron thus occurs as an adaptive response to nephron loss; however, this can lead to glomerular hypertension, glomerulosclerosis, and a progressive decline in kidney function [12].

Nephron number can be estimated by kidney biopsy together with careful direct radiographic measurements of nephron mass [13]. Obviously, this is not a routine clinical tool. Fortunately, there are strong data that show a good correlation between kidney volume and nephron number in infants [14, 15]. In humans, while the neonatal consequences of prematurity have been described [4, 6, 16], its long-term effects on kidney function during childhood remain to be determined. To bridge this gap in knowledge, we carried out a study to determine the impact of prematurity on kidney development in a cohort of extremely preterm infants from term corrected (37 postmenstrual age) until age 24 months.

## Methods

This prospective study was conducted in the Department of Neonatology, Townsville Hospital, Queensland, Australia. The recruitment was conducted from August 2014 until October 2016, and follow-up was completed in October 2018. We have previously published the neonatal outcomes [16], and the data presented in this manuscript are follow-up data from the same cohort. Preterm infants at less than 28 weeks of gestation (extremely preterm infants), with birth weights between the 10th and 90th centile (appropriate for gestational age (AGA)), and admitted to the neonatal department during the study period were followed from term corrected (37 postmenstrual age (PMA)) upon discharge from the neonatal unit at ages of 6, 12, and 24 months. A cohort of term AGA infants admitted to the neonatal unit at the same time with minor neonatal conditions, such as jaundice or feeding problems, were recruited as a control group. The preterm infants were compared with a control group of similar corrected gestational age, to ensure that any differences found were not due to prematurity. Infants diagnosed with antenatal kidney abnormalities as reported by a radiologist were excluded.

The Townsville Health District Human Research Ethics Committee approved this study, which was conducted following the tenets of the Declaration of Helsinki. Written consent was obtained from parents of all infants who participated in this study. The primary outcomes from this study were total kidney volume (TKV) (a surrogate for nephron number) and estimated glomerular filtration rate (eGFR). Secondary outcomes were albuminuria and mean blood pressure measurements.

The methods used to determine kidney volume and other measurements (cystatin C and urine for albumin to creatinine ratio and statistical analysis) have been previously published [16]. Estimated glomerular filtration rate was calculated using the Zappitelli CysC eGFR equation (GFR (mL/min per 1.73 m^2^) = 75.94/[serum cystatin C^1.17^]) [17]. Body surface area (BSA) was calculated using the Du Bois formula [18], which was then used to calculate the TKV/BSA ratio.

Blood pressure measurement was performed using a portable device (Welch Allyn Connex 3400 SureBP, Hillrom, Chicago, USA) with an appropriate cuff size and the mean blood pressure (MAP) was recorded. The infant usually sits on the parent’s lap, and the measurements are taken twice (when the infant is calm) for an average measurement. MAP is the commonly used blood pressure indicator in a neonatal unit, and it represents the average perfusion pressure. A single MAP value is easier to trend; changes in MAP are easier to interpret than changes in systolic or diastolic pressures, which at times can move in different directions [19].

## Results

**Fig. 1:**
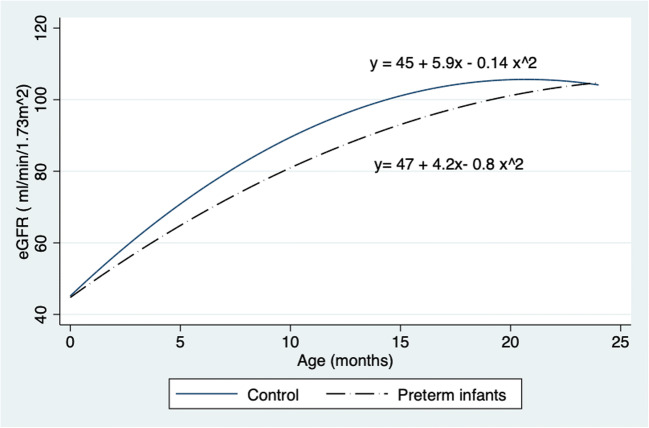
Fitted quadratic regression curve for eGFR (mL/min/1.73m^2^) in preterm and control infants in the first 2 years of life

**Fig. 2:**
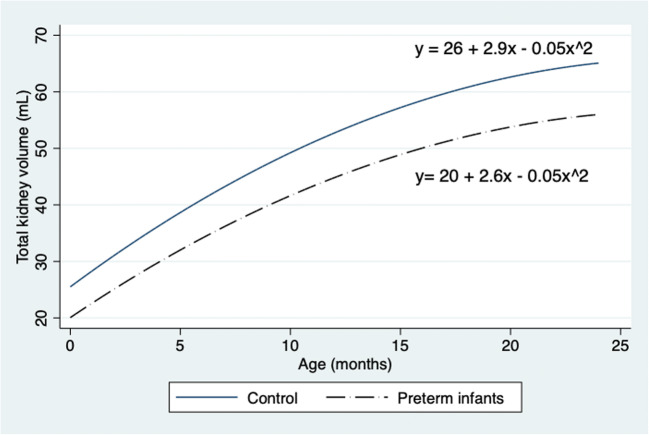
Fitted quadratic regression curves comparing total kidney volumes (TKV) between preterm infants and controls

**Fig. 3:**
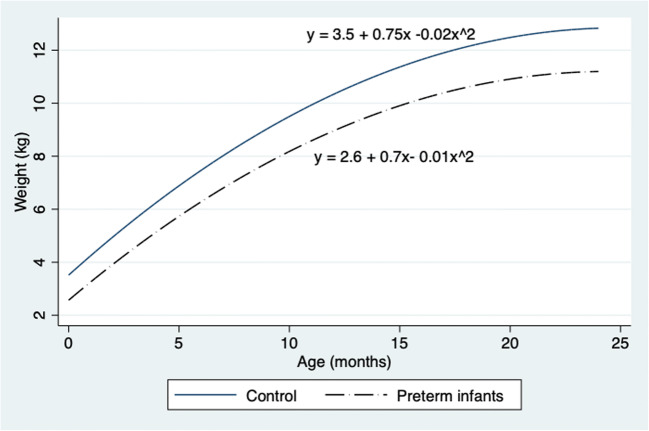
Fitted quadratic regression curve comparing body weights between preterm infants and controls

During the study, 131 preterm infants less than 28 weeks of gestation were admitted to the neonatal unit. There were nine deaths. Consent was obtained for 59 infants, and one infant was excluded because of hydronephrosis. Complete data sets were available for 53 infants; 32 term infants were recruited as the control group. All infants were followed up at 6, 12, and 24 months after discharge from the neonatal unit. At 24 months, infants born preterm had significantly smaller TKVs (56.1 (9.4) vs. 64.8 (10.2) mL; *P* = 0.006) although there was no difference in eGFR (102.1 (14.9) vs.104.6 (21.5) mL/min/1.73 m^2^; *P* = 0.7). These infants were smaller (11.25 (1.53) vs.12.9 (1.8) kg; *P* = 0.002) and shorter (83.8 (3.0) vs. 86.3 (3.4) cm; *P* = 0.02) compared with the control group. There were no significant differences in mean blood pressure (80.5 (11.0) vs. 75.6 (9.5) mm Hg) and urine albumin to creatinine ratio between preterm and control groups respectively. Table 1 summarizes the comparisons between infants born preterm and the control group at 6, 12, and 24 months. eGFR in both groups had plateaued by 24 months of age, as shown in Fig. 1, although differences in TKVs and body weights were still evident, as shown in Figs. 2 and 3 respectively. When corrected for BSA, there was no difference in TKV (TKV/BSA) between premature and term neonates (112 (14) vs. 120 (16) mL/m^2^; *P* = 0.09). However, when corrected for length (TKV/length), preterm infants had significantly smaller kidney volume (0.54 (0.1) vs. 0.59 (0.1) mL/cm, *P* = 0.05; 0.61 (0.1) vs. 0.71 (0.1) mL/cm, *P* = 0.003; and 0.67 (0.1) vs. 0.76 (0.1) mL/cm, *P* = 0.006) at 6, 12, and 18 months respectively.

**Table 1:** Comparison between infants born preterm and control at 6, 12, and 24 months of age

	6 months			12 months			24 months		
	Preterm	Control (term)	*P* value	Preterm	Control (term)	*P* value	Preterm	Control (term)	*P* value
Weight (kg)	6.6 (1.1)	7.9 (1.0)	< 0.001*	8.7 (1.3)	10.0 (1.1)	< 0.001*	11.2 (1.5)	12.9 (1.8)	0.002*
Length (cm)	64.1 (2.9)	67.5 (3.1)	< 0.001*	73.1 (3.5)	75.6 (2.9)	0.01*	83.8 (3.0)	86.3 (3.4)	0.02*
Total kidney volume (mL)	34.5 (7.3)	39.8 (7.4)	0.006*	44.5 (9.4)	53.8 (10.4)	0.001*	56.1 (9.4)	64.8 (10.2)	0.01*
eGFR (mL/min/1.73 m^2^)	69.2 (14.4)	77.1 (12.4)	0.04*	85.5 (13.3)	93.1 (20.2)	0.12	102. 1 (14.9)	104.6 (21.5)	0.70

*eGFR*, estimated glomerular filtration rate

*Statistically significant difference (*P* < 0.05)

**Fig. 4:**
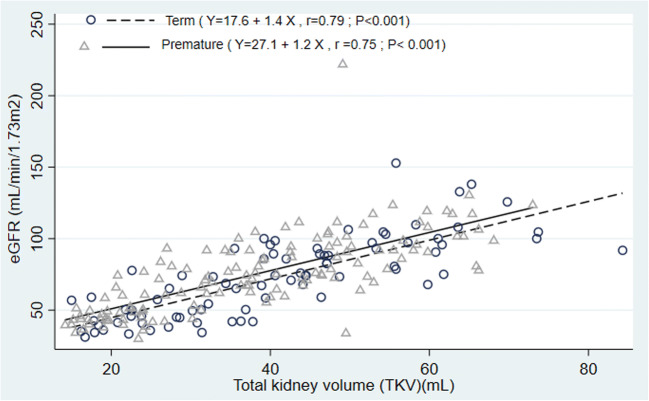
Premature and control cohorts show significant positive (Pearson’s) correlation between TKV and eGFR in the first 24 months

Figure 4 shows that TKV in both premature and control cohorts shows significant positive (Pearson’s) correlation between TKV and eGFR in the first 24 months.

## Discussion

Infant GFR peaks by the age of 2 years [20, 21]; our data for both term and preterm infants show a similar trend in Fig. 1. Height-adjusted TKVs in premature infants were significantly smaller when compared with the control group. Height-adjusted TKVs have been found to be useful in predicting the onset of kidney insufficiency in certain forms of cystic kidney disease [22]. We suggest that they may be more useful than TKV to BSA ratios for assessing kidney development in premature infants. Despite having smaller kidney volumes (hence reduced nephron numbers), preterm infants had eGFRs similar to those measured in infants born at term (control). We propose that preterm infants achieve an eGFR similar to term infants despite having fewer nephrons probably by single-nephron hyperfiltration, thus putting these infants on a trajectory to develop CKD [23, 24]. As nephrogenesis occurs in utero, the increase in eGFR as shown in Fig. 4 is likely to be due to an increase in kidney blood flow, glomerulomegaly, and an increase in kidney tubular length (the last two result in an increased TKV).

Keijzer-Veen et al. [25] assessed kidney function and kidney size in a cohort of 29 young adults (20 years old) who were born preterm (< 32 weeks’ gestation). They did not find any difference in kidney function and kidney size between the preterm and control groups. However, more recent studies involving extremely preterm infants (born earlier at < 28 weeks gestation) show a different trend. Rakow et al. [26] followed up a cohort of 40 extremely preterm (< 28 weeks) infants until the age of 7 years. The investigators found that at 7 years of age, these children had significantly smaller kidney volumes but there were no significant differences in their eGFRs or blood pressures. In another study, Vollsaeter et al. [27] published data, collected at age 11 years, from a cohort of 57 children who were also born extremely preterm (< 28 weeks). In this study, the investigators reported that eGFRs in these children were significantly lower when compared with the control (term) group. There was no significant difference in blood pressure. More recently, South et al. [28] published follow-up data from a cohort of 96 adolescents (14 years) who were born preterm (< 28 weeks) and showed that these children had high blood pressures and lower eGFRs compared with their peers who were born at term. Our study follows extremely preterm infants of similar gestational age (< 28 weeks) as the above three studies and it is likely that the impact of prematurity on nephrogenesis is more profound in smaller and more preterm infants, especially those < 28 weeks of gestation. We postulate that preterm infants have glomerular hyperfiltration within the first week of life [16], and this continues during infancy and childhood, so that they show signs of kidney impairment by puberty and adolescence [27, 28]. It is plausible that the increase in GFR after puberty cannot keep up with the growth surges a child experiences during puberty and its associated increased metabolic demand and hormonal changes, which eventually lead to high blood pressure.

Most preterm infants’ follow-up plans involve regular assessment until the age of 24 months, which is followed by a formal neurodevelopmental assessment. These infants often have their weight, length, and head circumference measured [29]. Kidney function as measured by urine analysis for proteinuria, blood pressure, and eGFR using cystatin C are not part of their routine assessment. We recommend that for infants born < 28 weeks gestation these parameters of kidney function are routinely measured at 2 years of age, and at adolescence when the first signs of kidney impairment may become clinically evident.

Our study has a few limitations. Firstly, our sample size was limited, especially for the control group. Approximately 40% of the patients in our cohort are from rural and regional areas and were not keen/able to travel to the study site for regular assessment, so six subjects were lost to follow-up. It is rather challenging to have parents of normal infants attend regular follow-ups and to subject the infants to regular venepuncture, when it is not clinically indicated. So, we are very grateful to all parents who willingly enrol their infant for clinical studies. In our study, we used eGFR to determine kidney function, using Zappitelli’s equation [17]. Regular GFR measurement in children using the inulin method is not practical in the clinical setting. We used the Du Bois method to estimate BSA area, and this is only an approximation of the actual BSA. While the sonographer was blinded to the clinical data, it is always possible for the sonographer to guess this clinical status (preterm or term) based on other parameters such as plagiocephaly, which occurs in preterm infants.

## Conclusion

Relative to body length, TKVs in premature infants are smaller. Despite having smaller kidney volumes (and therefore reduced nephron numbers), preterm infants have an eGFR similar to term infants (control). There was no difference in blood pressure and the urine albumin to creatinine (ACR) ratios between the two cohorts. Preterm infants achieve similar eGFRs to term infants, probably by single-nephron hyperfiltration. This puts them on a trajectory that could lead to intraglomerular hypertension and glomerulosclerosis. Follow-up of preterm infants should include routine assessment of the kidney function (eGFR and ACR) and blood pressure.
